# The efficacy of immunotherapy and chemoimmunotherapy in patients with advanced rare tumors: A Turkish oncology group (TOG) study

**DOI:** 10.1002/cam4.6869

**Published:** 2023-12-22

**Authors:** Deniz Can Guven, Musa Baris Aykan, Harun Muglu, Ertugrul Bayram, Kaan Helvaci, Bengü Dursun, Melisa Celayir, Elvin Chelebiyev, Erdinc Nayir, Mustafa Erman, Ahmet Sezer, Yuksel Urun, Umut Demirci, Ozlem Er, Umut Disel, Ahmet Bilici, Cagatay Arslan, Nuri Karadurmus, Saadettin Kilickap

**Affiliations:** ^1^ Department of Medical Oncology Hacettepe University Cancer Institute Ankara Turkey; ^2^ Department of Medical Oncology Gulhane School of Medicine, University of Health Sciences Ankara Turkey; ^3^ Istanbul Medipol University Faculty of Medicine Istanbul Turkey; ^4^ Department of Medical Oncology Cukurova University Adana Turkey; ^5^ Memorial Ankara Hospital Ankara Turkey; ^6^ Department of Medical Oncology Ankara University Ankara Turkey; ^7^ Department of Medical Oncology MAA Acıbadem University İstanbul Turkey; ^8^ Department of Medical Oncology Mersin Medical Park Hospital Mersin Turkey; ^9^ Baskent University Adana Hospital Adana Turkey; ^10^ Department of Medical Oncology Acibadem Adana Hospital Adana Turkey; ^11^ Department of Medical Oncology School of Medicine, Medical Park Hospital, Izmir Economy University Izmir Turkey; ^12^ Department of Medical Oncology Istinye University Faculty of Medicine Istanbul Turkey

**Keywords:** immune checkpoint inhibitor, immunotherapy, rare tumor, sarcoma

## Abstract

**Introduction:**

The advances in immune checkpoint inhibitors (ICIs) were relatively slow in rare tumors. Therefore, we conducted a multi‐center study evaluating the efficacy of ICI monotherapy and the combination of ICIs with chemotherapy (CT) in patients with advanced rare tumors.

**Methods:**

In this retrospective cohort study, we included 93 patients treated with ICIs for NCI‐defined rare tumors from the 12 cancer centers in Turkey. The primary endpoints were the overall response (ORR) and disease control rate (DCR).

**Results:**

The cohort's median age was 56, and 53.8% of the patients were male. The most frequent diagnosis was sarcoma (29%), and 81.7% of the patients were previously treated with at least one line of systemic therapy in the advanced stage.

The ORR and DCR were 36.8% and 63.2%, respectively. The germ cell tumors had the lowest ORR (0%), while the Merkel cell carcinoma had the highest ORR to ICIs (57.1%). Patients treated with ICI + ICI or ICI plus chemotherapy combinations had higher ORR (55.2% vs. 27.6%, *p* = 0.012) and DCR (82.8% vs. 53.4%, *p* = 0.008).

The median OS was 13.47 (95% CI: 7.79–19.15) months, and the six and 12‐month survival rates were 71% and 52%. The median duration of response was 16.59 months, and the 12‐month progression‐free survival rate was 66% in responders. The median time‐to‐treatment failure was 5.06 months (95% CI: 3.42–6.71). Three patients had high‐grade irAEs with ICIs (grade 3 colitis, grade 3 gastritis, and grade 3 encephalitis in one patient each).

**Conclusion:**

We observed over 30% ORR and a 13‐month median OS in patients with rare cancers treated with ICI monotherapy or ICI plus CT combinations. The response rates to ICIs or ICIs plus CT significantly varied across different tumor types. Responding patients had over 2 years of survival, highlighting a need for further trials with ICIs for patients with rare tumors.

## 1 INTRODUCTION

Immune checkpoint inhibitors (ICIs) became an indispensable part of the oncology practice for almost all patients with advanced cancers,^1^ including but not limited to melanoma,^2^ renal cell carcinoma (RCC),^3^ non‐small cell lung cancer (NSCLC),^4^ gastric cancer^5^ and Hodgkin lymphoma (HL).^6^ Furthermore, the ICIs entered into the adjuvant or neoadjuvant in several tumors.^7^, ^8^, ^9^ However, the advances in ICIs were not uniform for all tumors. The available trials mainly focused on tumors with an active immune milieu^10^ or tumors with a higher incidence or prevalence, while the interest and advances in the ICI field were relatively slow in most rare tumors.^11^

Rare tumors are a significant but understudied problem.^12^ While definitions vary across organizations, the NCI defines rare tumors as tumors with an incidence of 15 or fewer cases for 100,000 people per year.^13^ While individually rare, the rare cancers constitute almost over 20% of newly diagnosed cancers.^14^ However, the developments in rare cancers were slow due to problems with case definition and diagnosis, limitations with clinical trial involvement, and lesser support from the industry due to a smaller target sample size.^11^, ^14^, ^15^ Additionally, the relative inefficacy of ICIs in earlier trials of sarcoma^16^ and neuroendocrine tumors^17^ further slowed the interest in ICI use in rare tumors. However, ICIs changed the fortunes of patients with several rare tumors like Merkel cell carcinoma (MCC)^18^ and Kaposi sarcoma (KS).^19^ Furthermore, a recent phase II basket trial demonstrated clinical efficacies at least similar to chemotherapy in the same treatment setting with a low rate of high‐grade adverse events.^20^ These issues define the need for further delineation of the ICI efficacy in rare cancers.

In addition to the limited clinical trial data, real‐life data with ICIs in rare cancers is even more scarce. However, the ICIs could be used for patients with rare cancers, especially in the later treatment lines, due to limited therapeutic options. Similar to the basket trials in rare cancers, evaluating ICI efficacy in real‐life basket cohorts is paramount to finding patient groups garnering a significant benefit with ICIs and preventing some patients from relatively ineffective treatments. Due to the rarity of the individual rare tumors, multi‐center studies including several rare cancers, could be a feasible way to gather high‐quality and comprehensive data. Based on these reasons, we conducted a multi‐center study evaluating the ICI efficacy in NCI‐defined rare tumors without a phase III study.

## 2 METHODS

### 2.1 Study cohort

We included patients treated with ICIs for NCI‐defined rare cancers between January 2016 to December 2021 from the 12 comprehensive cancer centers in Turkey. We included the patients independent of the biomarker status, the ICI type, and the use of CT combined with ICI and treatment line. We excluded patients with the missing significant clinical data and those who lost to the follow‐up. All patients reached the treatment the out of pocket or via private insurance. We recorded the following variables: Baseline demographics, height and weight, comorbidities, ICI and tumor type, Eastern Cooperative Oncology Group (ECOG) performance status (PS), sites of metastasis, number of previous systemic treatments, microsatellite status, next‐generation sequencing findings, the best response to ICIs, the times of first ICI dose, progression, and last follow‐up, and high‐grade immune‐related adverse events (irAEs) under treatment. The ICI response was extracted from the previously retrieved imaging reports evaluated according to RECIST 1.1 criteria,^21^ and irAEs were classified according to CTCAE version 5.^22^

### 2.2 Statistical analyses

We expressed the baseline characteristics with medians and interquartile ranges (IQR) for continuous variables and frequencies and percentages for categorical variables. The primary endpoints were the overall response (ORR) and disease control rate (DCR). The survival outcomes and adverse events were the secondary endpoints. The progression‐free survival (PFS) was defined as the time from ICI commencing to the time of progression or death, and the overall survival (OS) was defined as the time from ICI beginning to the time of death. In addition to PFS and OS, the time‐to‐treatment‐failure (TTF) was evaluated as recommended in the real‐world cohorts.^23^ The TTF was defined as treatment discontinuation before 2 years for any reason, including cancer progression, adverse events, patient choice, or death. The follow‐up time was calculated with the reverse Kaplan–Meier method. The univariate survival analyses were conducted with Kaplan–Meier survival curves, and survival analyses across subgroups were conducted with the log‐rank test. The association of clinical parameters with ORR and DCR was evaluated with chi‐square and Fischer's exact tests. We performed statistical analyses with SPSS, version 25.0 (IBM Inc., Armonk, NY, USA), and considered a type error level of 5% (*p* < 0.05) as the threshold limit for statistical significance.

## 3 RESULTS

### 3.1 Baseline characteristics

A total of 93 patients with NCI‐defined rare tumors treated with ICIs and had adequate clinical data were included in the study. The median age of the study cohort was 56 (IQR 33–66), and 53.8% of the patients were male. 60.2% of the patients had no comorbidities, and 37.6% had ECOG PS of zero. Soft tissue sarcoma (17.2%), rare head and neck cancers (HNC) (14%), and bone sarcoma (11.8%) were the most frequent diagnoses. 59.1% of the patients had more than one site of metastasis, and lung metastases were the most prevalent metastatic disease site (52.7%). Nivolumab was the most frequently used ICI (46.2%), and 81.7% of the patients were previously treated with at least one line of systemic therapy in the advanced stage before ICIs. 28% of the patients were treated with ICI and chemotherapy (CT), and 4.3% were treated with ICI + ICI combinations. The ICI plus chemotherapy combinations were frequently used in patients with carcinoma of unknown primary (80%) and neuroendocrine tumors (75%). In comparison, the ICI plus CT use was less than 30% in the remaining rare tumor types [soft tissue sarcoma (6.7%), rare genitourinary tumors (16.7%), and thymic tumors (14.3%)]. The baseline characteristics of the patients are summarized in Table 1.

**TABLE 1:** Baseline characteristics of the study cohort.

Clinical feature	*n* (%)
Sex	
Male	50 (53.8)
Female	43 (46.2)
Comorbidities	
Absent	56 (60.2)
Present	37 (39.8)
ECOG PS	
0	35 (38.5)
1	37 (40.7)
2	14 (15.4)
3	4 (4.4)
4	1 (1.1)
Immunotherapy agent	
Nivolumab	43 (46.2)
Pembrolizumab	28 (30.1)
Atezolizumab	16 (17.2)
Nivolumab + Ipilimumab	4 (4.3)
Avelumab	2 (2.2)
Combination therapy	
Absent	63 (67.7)
Present	30 (32.3)
Primary tumor	
Soft Tissue Sarcoma	16 (17.2)
Rare HNC	13 (14)
Bone Sarcoma	11 (11.8)
CUP	9 (9.7)
NET/NEC	8 (8.6)
MCC and Skin Cancers	7 (7.5)
Other	29 (31.2)
Line of treatment	
1	17 (18.3)
2	31 (33.3)
3	19 (20.4)
4 or later	26 (28)
Metastatic sites	
1	38 (40.9)
2	26 (28)
3 or more	29 (31.1)

Abbreviations: CUP, carcinoma of unknown primary; HNC, head and neck cancer; MCC, Merkel cell carcinoma; NEC, neuroendocrine carcinoma; NET, neuroendocrine tumor.

### 3.2 Efficacy evaluation

The patients were given a median of 6 (IQR 4–11) ICI infusions, and the median follow‐up was 24.38 (IQR 8.87–32.10) months. The radiologic response was evaluable for 87 of 93 patients. The complete and partial responses were seen in 6.9% and 29.9% of the patients, respectively. The ORR and DCR were 36.8% and 63.2%. The GCT had the lowest ORR (0%), while the MCC had the highest ORR to ICIs (57.1%). The ORR and DCR according to tumor type are summarized in Table 2. Among soft tissue sarcoma subtypes, responses were recorded in patients with angiosarcoma (1/1), dendritic cell sarcoma (1/1), KS (1/1), leiomyosarcoma (1/6) and unclassified sarcoma (1/3). Among bone sarcomas, only patients with Ewing sarcoma had a radiological response to ICIs or ICIs plus CT, while no response was recorded in patients with osteosarcoma, chondrosarcoma, or chordoma (Supplementary Table 1).

**TABLE 2:** The overall response rate (ORR) and disease control rate (DCR) according to tumor type.

	ORR		DCR	
	Absent	Present	Absent	Present
	*n* (%)	*n* (%)	*n* (%)	*n* (%)
Tumor type				
Soft Tissue Sarcoma	11 (68.7)	5 (31.3)	9 (56.3)	7 (43.7)
Thymic Tumor	4 (80)	1 (20)	2 (40)	3 (60)
CUP	5 (55.6)	4 (44.4)	0 (0)	9 (100)
GCT	4 (100)	0 (0)	2 (50)	2 (50)
Rare HNC	5 (45.5)	6 (55.5)	3 (27.2)	8 (72.8)
NET/NEC	4 (50)	4 (50)	2 (25)	6 (75)
Other	5 (71.4)	2 (28.6)	2 (28.6)	5 (71.4)
Bone Sarcoma	8 (72.8)	3 (27.2)	6 (55.5)	5 (45.5)
MCC and Skin Cancer	3 (42.9)	4 (57.1)	2 (28.6)	5 (71.4)
Hepatobiliary	2 (66.7)	1 (33.3)	2 (66.7)	1 (33.3)
Rare GU	4 (66.7)	2 (33.3)	2 (33.3)	4 (66.7)
Total	55 (63.2)	32 (36.8)	32 (36.8)	55 (63.2)

*Note*: ORR: the presence of complete or partial response to treatment, DCR: the presence of complete response, partial response or stable disease with treatment.

Abbreviations: CUP, carcinoma of unknown primary; GU, genitourinary; HNC, head and neck cancer; MCC, Merkel cell carcinoma; NEC, neuroendocrine carcinoma; NET, neuroendocrine tumor.

The association of ORR and sex, treatment line (1st or 2nd vs. later lines), baseline liver metastases, LDH levels (N vs. >ULN), and ECOG (0 vs. 1 or higher) did not reach statistical significance (*p* > 0.05 for each). Most of these clinical parameters (sex, ECOG PS, LDH levels, and liver metastasis) did not have a statistically significant association with DCR rates. Patients treated with ICI + ICI or ICI plus chemotherapy combinations had higher ORR (55.2% vs. 27.6%, *p* = 0.012) and DCR (82.8% vs. 53.4%, *p* = 0.008) (Table 3). The 66.7% of the patients treated with ICI‐ICI or ICI plus chemotherapy combinations were treated with these combinations in the first or second‐line treatment. Additionally, patients treated in the earlier lines (1st or 2nd vs. later lines) had higher DCR with ICIs (76.7% vs. 50%, *p* = 0.010).

**TABLE 3:** The association between clinical parameters and overall response rate (ORR) and disease control rate (DCR).

	ORR (*n*, %)	*p*‐value	DCR (*n*, %)	*p*‐value
Age group				
<65 years of age	20 (31.7)	0.115	36 (57.1)	0.057
>65 years of age	12 (50%)		19 (79.2)	
Sex				
Female	13 (33.3)	0.548	25 (64.1)	0.877
Male	19 (39.6)		30 (62.5)	
Line of treatment				
1st or 2nd line	19 (44.2)	0.157	33 (76.7)	**0.010**
3rd line or later	13 (29.5)		22 (50)	
Combination therapy (ICI + ICI or ICI + CT)				
Monotherapy	16 (27.6)	**0.012**	31 (53.4)	**0.008**
Combination	16 (55.2)		24 (82.8)	
LDH levels				
Normal	21 (45.7)	0.080	32 (69.6)	0.208
>ULN	9 (26.5)		19 (55.9)	
Liver metastasis				
Absent	25 (39.1)	0.462	44 (68.8)	0.074
Present	7 (30.4)		11 (47.8)	
Total number of metastatic sites				
1 or 2	24 (40.7)	0.274	39 (66.1)	0.418
3 or more	8 (28.6)		16 (57.1)	
ECOG				
0	11 (34.4)	0.755	19 (59.4)	0.660
1 or higher	20 (37.7)		34 (64.2)	

*Note*: ORR: the presence of complete or partial response to treatment, DCR: the presence of complete response, partial response or stable disease with treatment. Bold values denote statistical significance.

Abbreviations: CT, chemotherapy; ICI, immune checkpoint inhibitor; ULN, upper limit of normal.

**FIGURE 1:**
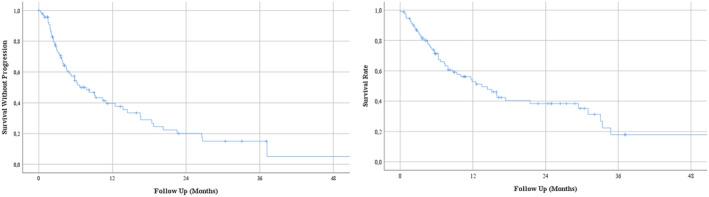
The Kaplan–Meier curves for progression‐free survival (left) and overall survival (right).

**FIGURE 2:**
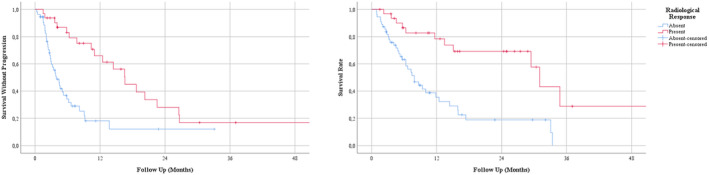
Progression‐free survival (left) and overall survival (right) according to the presence of radiological response.

The median PFS and OS were 7.72 (95% CI: 4.69–10.75) and 13.47 (95% CI: 7.79–19.15) months, respectively (Figure 1). The six and 12‐month survival rates were 71% and 52%. The median duration of response was 16.59 months (95% CI: 12.54–20.64), and the 12‐month PFS rate was 66% in responders. The median TTF was 5.06 months (95% CI: 3.42–6.71).The OS (31.01 vs. 7.89, *p* < 0.001) and PFS (16.59 vs. 3.91 months, <0.001) were significantly longer in patients with a complete or partial response to ICIs than the patients with stable disease or progressive disease (Figure 2). Similarly, the presence of disease control was associated with longer PFS (13.73 vs. 2.43, *p* < 0.001) and OS (29.44 vs. 6.31, *p* < 0.001). Patients with liver metastasis at baseline had shorter OS (6.67 vs. 15.97 months, *p* < 0.001) and PFS (4.60 vs. 9.20 months, *p* = 0.012). Additionally, patients with higher LDH levels (>ULN) had shorter OS (7.43 vs. 29.44 months, *p* = 0.049) compared to patients with normal LDH levels at baseline (Figure 2). The association with OS and treatment line (first and second line vs. later lines, *p* = 0.652), and ECOG PS (*p* = 0.065) did not reach statistical significance. The PFS analyses were consistent with OS analyses other than a lack of statistically significant association between LDH levels and PFS (*p* = 0.188).

Three patients had high‐grade irAEs (grade 3 colitis, grade 3 gastritis, and grade 3 encephalitis in one patient each). All three patients were treated with steroids. The irAEs were resolved in two patients without sequela. The patient with grade 3 encephalitis also had grade 2 hepatitis. The encephalitis partly resolved in this patient, and ICI was permanently discontinued.

A total of nine patients were treated with ICIs according to biomarker selection. Five patients had microsatellite instability‐high (MSI‐H) tumors, and four patients had high tumor mutational burden (TMB) (>10 mutation/megabase). The ORR and DCR were 33.3% and 77.7% in this cohort. A patient with osteosarcoma with high TMB and a patient with MSI‐H rhabdomyosarcoma had progressive disease as the best response to ICIs.

## 4 DISCUSSION

In this multi‐center rare cancer cohort, we observed that ICI was associated with 36.8% ORR. The response rates were higher in patients with MCC, rare HNC, and CUP, while patients with GCT, and bone sarcoma had lower response rates to ICIs. The ORR was higher in patients treated with ICI + ICI or ICI plus chemotherapy combinations. Patients who responded to ICIs had over 2‐years of median survival with ICIs. The ICIs were generally safe and tolerable.

We observed 31% ORR in patients with soft tissue sarcoma. This figure was higher with the previous phase I/II trials reporting ORRs varied between 0% and 19%.^24^, ^25^ In the pooled analysis of the phase II sarcoma trials, the ORR was 15.1% with ICI monotherapy.^16^ However, there was considerable efficacy in patients with angiosarcoma,^26^ alveolar soft part sarcoma,^27^ and undifferentiated pleomorphic sarcoma^28^ in clinical trials. In addition to these tumors, patients with KS garnered a significant benefit in a recent phase II study of pembrolizumab.^19^ We observed responses in patients with angiosarcoma, follicular dendritic cell sarcoma, malignant mesenchymal tumor, LMS, and KS in our cohort. In contrast to sarcomas with a predilection to response to ICIs, patients with LMS had anecdotal responses to ICIs in clinical trials.^24^ We observed only one response in seven patients with LMS in our cohort. In addition to these clinical trials, Monga et al. retrospectively reviewed the ICI efficacy in a multicenter cohort of four institutions (*n* = 88). The authors reported a 23.8% ORR and a PFS of 4.1 months.^29^ Interestingly, the study reported a 45% ORR in patients with LMS, contrasting our study and previous clinical trials. Groisberg et al. retrospectively analyzed the outcomes of the 50 patients with sarcoma enrolled in the early phase immunotherapy trials. The authors reported a median OS of 13.4 months, although the ORR was only 4% in the cohort.^30^

Patients with GCT had very limited benefit from ICIs in the early phase clinical trials, and two small phase II trials reported no responses to ICIs.^31^, ^32^ While the sample size was small, we observed no response in patients with GCT treated with ICIs. Although the germ cell tumors have a rich immune infiltrate in tumor microenvironment,^33^ the low TMB^34^ and low levels of PD‐1 expression^35^ could be among the reasons for limited ICI efficacy in patients with GCTs.

The ICI efficacy was limited in our cohort in patients with bone sarcoma, similar to the previous clinical trials.^28^ However, three patients with Ewing sarcoma had responses to ICIs. Two of these patients were treated with combination therapy (chemotherapy + ICI and ICI + ICI in one each), and the other patient had MSI‐H disease. No response was observed in monotherapy trials with Nivolumab^36^ and Atezolizumab,^37^ a patient with Ewing sarcoma had a radiological response to nivolumab plus ipilimumab in the combination arm of ADVL1412 study.^38^ In a real‐world retrospective cohort, Scheinberg et al. evaluated the efficacy of ICIs in 18 adolescents and young adults with soft tissue or bone sarcomas. The authors reported radiological responses in one Ewing sarcoma patient among ten bone sarcoma patients.^39^ While it should be noted that the clinical trials of Vigil immunotherapy, an autologous tumor cell therapy, seem more promising,^40^ the ICI‐based combinations should be further evaluated in patients with Ewing sarcoma.

Patients with CUP have very limited therapeutic options and have a poor prognosis.^41^, ^42^ The ICIs could create another option for patients with CUP, either alone or in combination with chemotherapy.^43^ Raghav et al. recently reported a 20% ORR in patients with CUP treated with pembrolizumab in a phase II trial.^44^ We observed higher figures than this data, possibly due to the use of concomitant chemotherapy and treatment of patients in the first‐line mostly. We think that our data support the exploitation of ICI efficacy in patients with CUP in earlier settings and with chemotherapy.

Tumor profiling and molecular matched therapy emerged as the new approach for cancer therapy in the last decade with the advances in precision medicine.^45^, ^46^ While tumor profiling for matched therapy was feasible in the available trials, the exact benefit of this approach is yet to be defined for most tumors.^47^, ^48^ However, patients with rare cancers should be considered as early as possible for matched therapy due to the limited treatment options in most cases. A small percentage of our cohort was treated with ICIs according to tumor molecular profiling. The DCR was promising (77.7%) in these patients, and this cohort included rare tumors with very limited options, like bladder squamous cell carcinoma, anaplastic glioneuronal tumor, and ACC.

The present study is subject to several limitations inherent to retrospective design and patient cohort. A modest number of patients in subgroups prevented us from conducting additional subgroup analyses and reaching definitive conclusions, and made our results mostly hypothesis‐generating. Most of our patients were treated in the later lines and in a biomarker unselected manner, limiting the generality of our results to patients treated in the countries with access to immunotherapy in the earlier lines and molecular profiling. However, despite these limitations, we conducted a large‐scale study on ICI efficacy in patients with rare cancers, an area with significant unmet need. Our study adds to the limited body of evidence regarding the efficacy of ICIs in real‐life cohorts.^29^, ^30^, ^39^, ^49^

We observed promising response and disease control rates with ICIs in patients with rare tumors. The response rates were higher; the ICIs were used in combination with chemotherapy or with ICI‐ICI combinations. While we are waiting for more prospective evidence, our observations support the use of ICIs for patients with rare tumors could be a pragmatic approach for patient benefit.

## AUTHOR CONTRIBUTIONS

**Deniz Can Guven:** Conceptualization (equal); data curation (lead); formal analysis (lead); investigation (equal); methodology (equal); project administration (equal); writing – original draft (lead). **Musa Baris Aykan:** Data curation (equal); investigation (equal). **Harun Muglu:** Data curation (equal); investigation (equal). **Ertugrul Bayram:** Data curation (equal); investigation (equal). **Kaan Helvaci:** Data curation (equal); investigation (equal). **Bengü Dursun:** Data curation (equal); investigation (equal). **Melisa Celayir:** Data curation (equal); investigation (equal). **Elvin Chelebiyev:** Data curation (equal); investigation (equal). **Erdinc Nayir:** Data curation (equal); investigation (equal). **Mustafa Erman:** Data curation (equal); investigation (equal); writing – review and editing (equal). **Ahmet Sezer:** Supervision (equal); writing – review and editing (equal). **Yuksel Urun:** Supervision (equal); writing – review and editing (equal). **Umut Demirci:** Supervision (equal); writing – review and editing (equal). **Ozlem Er:** Supervision (equal); writing – review and editing (equal). **Umut Disel:** Resources (supporting); supervision (supporting); writing – review and editing (supporting). **Ahmet Bilici:** Supervision (equal); writing – review and editing (equal). **C. Arslan:** Supervision (equal); writing – review and editing (equal). **Nuri Karadurmus:** Supervision (equal); writing – review and editing (equal). **Saadettin Kilickap:** Conceptualization (equal); methodology (equal); supervision (lead); writing – review and editing (lead).

## FUNDING INFORMATION

The authors received no financial support for this article.

## CONFLICT OF INTEREST STATEMENT

The authors declare no conflict of interest.

## IRB APPROVAL

Ethical approval was granted by the Istinye University prior to commencing of the study.

## INFORMED CONSENT

Due to retrospective nature of the study, the need for informed consent was waived by IRB.

## Supporting information


Table S1.

## Data Availability

The data of this study is available from the corresponding author, upon reasonable request.

## References

[1] Shiravand Y , Khodadadi F , Kashani SM , et al. Immune checkpoint inhibitors in cancer therapy. Curr Oncol. 2022;29:3044‐3060. 35621637 10.3390/curroncol29050247 PMC9139602

[2] Wolchok JD , Chiarion‐Sileni V , Gonzalez R , et al. Long‐term outcomes with nivolumab plus ipilimumab or nivolumab alone versus ipilimumab in patients with advanced melanoma. J Clin Oncol. 2021;40:127‐137. 34818112 10.1200/JCO.21.02229 PMC8718224

[3] Powles T , Plimack ER , Soulières D , et al. Pembrolizumab plus axitinib versus sunitinib monotherapy as first‐line treatment of advanced renal cell carcinoma (KEYNOTE‐426): extended follow‐up from a randomised, open‐label, phase 3 trial. Lancet Oncol. 2020;21:1563‐1573. 33284113 10.1016/S1470-2045(20)30436-8

[4] Herbst RS , Giaccone G , de Marinis F , et al. Atezolizumab for first‐line treatment of PD‐L1‐selected patients with NSCLC. N Engl J Med. 2020;383:1328‐1339. 32997907 10.1056/NEJMoa1917346

[5] Janjigian YY , Shitara K , Moehler M , et al. First‐line nivolumab plus chemotherapy versus chemotherapy alone for advanced gastric, gastro‐oesophageal junction, and oesophageal adenocarcinoma (CheckMate 649): a randomised, open‐label, phase 3 trial. Lancet. 2021;398:27‐40. 34102137 10.1016/S0140-6736(21)00797-2 PMC8436782

[6] Chen R , Zinzani PL , Lee HJ , et al. Pembrolizumab in relapsed or refractory Hodgkin lymphoma: 2‐year follow‐up of KEYNOTE‐087. Blood. 2019;134:1144‐1153. 31409671 10.1182/blood.2019000324 PMC6776792

[7] Felip E , Altorki N , Zhou C , et al. Adjuvant atezolizumab after adjuvant chemotherapy in resected stage IB‐IIIA non‐small‐cell lung cancer (IMpower010): a randomised, multicentre, open‐label, phase 3 trial. Lancet. 2021;398:1344‐1357. 34555333 10.1016/S0140-6736(21)02098-5

[8] Forde PM , Spicer J , Lu S , et al. Neoadjuvant nivolumab plus chemotherapy in resectable lung cancer. N Engl J Med. 2022;386:1973‐1985. 35403841 10.1056/NEJMoa2202170 PMC9844511

[9] Choueiri TK , Tomczak P , Park SH , et al. Adjuvant pembrolizumab after nephrectomy in renal‐cell carcinoma. N Engl J Med. 2021;385:683‐694. 34407342 10.1056/NEJMoa2106391

[10] Hegde PS , Chen DS . Top 10 challenges in cancer immunotherapy. Immunity. 2020;52:17‐35. 31940268 10.1016/j.immuni.2019.12.011

[11] Guven DC , Stephen B , Sahin TK , Cakir IY , Erul E , Aksoy S . The efficacy of immune checkpoint inhibitors in rare tumors: a systematic review of published clinical trials. Crit Rev Oncol Hematol. 2022;174:103700. 35533815 10.1016/j.critrevonc.2022.103700

[12] Greenlee RT , Goodman MT , Lynch CF , Platz CE , Havener LA , Howe HL . The occurrence of rare cancers in US adults, 1995–2004. Public Health Rep. 2010;125:28‐43. 20402194 10.1177/003335491012500106 PMC2789814

[13] Alvi MA , Wilson RH , Salto‐Tellez M . Rare cancers: the greatest inequality in cancer research and oncology treatment. Br J Cancer. 2017;117:1255‐1257. 28934760 10.1038/bjc.2017.321 PMC5672935

[14] DeSantis CE , Kramer JL , Jemal A . The burden of rare cancers in the United States. CA Cancer J Clin. 2017;67:261‐272. 28542893 10.3322/caac.21400

[15] Komatsubara KM , Carvajal RD . The promise and challenges of rare cancer research. Lancet Oncol. 2016;17:136‐138. 26868336 10.1016/S1470-2045(15)00485-4

[16] Italiano A , Bellera C , D'Angelo S . PD1/PD‐L1 targeting in advanced soft‐tissue sarcomas: a pooled analysis of phase II trials. J Hematol Oncol. 2020;13:55. 32430039 10.1186/s13045-020-00891-5 PMC7236113

[17] Strosberg J , Mizuno N , Doi T , et al. Efficacy and safety of pembrolizumab in previously treated advanced neuroendocrine tumors: results from the phase II KEYNOTE‐158 study. Clin Cancer Res. 2020;26:2124‐2130. 31980466 10.1158/1078-0432.CCR-19-3014 PMC7811789

[18] D'Angelo SP , Bhatia S , Brohl AS , et al. Avelumab in patients with previously treated metastatic Merkel cell carcinoma: long‐term data and biomarker analyses from the single‐arm phase 2 JAVELIN Merkel 200 trial. J Immunother Cancer. 2020;8:e000674. 32414862 10.1136/jitc-2020-000674 PMC7239697

[19] Delyon J , Biard L , Renaud M , et al. PD‐1 blockade with pembrolizumab in classic or endemic Kaposi's sarcoma: a multicentre, single‐arm, phase 2 study. Lancet Oncol. 2022;23:491‐500. 35279271 10.1016/S1470-2045(22)00097-3

[20] Naing A , Meric‐Bernstam F , Stephen B , et al. Phase 2 study of pembrolizumab in patients with advanced rare cancers. J Immunother Cancer. 2020;8:8. 10.1136/jitc-2019-000347 PMC7078933 32188704

[21] Eisenhauer EA , Therasse P , Bogaerts J , et al. New response evaluation criteria in solid tumours: revised RECIST guideline (version 1.1). Eur J Cancer. 2009;45:228‐247. 19097774 10.1016/j.ejca.2008.10.026

[22] Institute NC . https://ctep.cancer.gov/protocolDevelopment/electronic_applications/docs/CTCAE_v5_Quick_Reference_8.5x11.pdf

[23] Sridhara R , Zhou J , Theoret MR , Mishra‐Kalyani PS . Time to treatment failure (TTF) as a potential clinical endpoint in real‐world evidence (RWE) studies of melanoma. J Clin Oncol. 2018;36:9578.

[24] Ben‐Ami E , Barysauskas CM , Solomon S , et al. Immunotherapy with single agent nivolumab for advanced leiomyosarcoma of the uterus: results of a phase 2 study. Cancer. 2017;123:3285‐3290. 28440953 10.1002/cncr.30738 PMC5762200

[25] Pollack SM , Redman MW , Baker KK , et al. Assessment of doxorubicin and pembrolizumab in patients with advanced anthracycline‐naive sarcoma: a phase 1/2 nonrandomized clinical trial. JAMA Oncol. 2020;6:1778‐1782. 32910151 10.1001/jamaoncol.2020.3689 PMC7489365

[26] Florou V , Rosenberg AE , Wieder E , et al. Angiosarcoma patients treated with immune checkpoint inhibitors: a case series of seven patients from a single institution. J Immunother Cancer. 2019;7:213. 31395100 10.1186/s40425-019-0689-7 PMC6686562

[27] Naqash AR , O'Sullivan Coyne GH , Moore N , et al. Phase II study of atezolizumab in advanced alveolar soft part sarcoma (ASPS). J Clin Oncol. 2021;39:11519.

[28] Tawbi HA , Burgess M , Bolejack V , et al. Pembrolizumab in advanced soft‐tissue sarcoma and bone sarcoma (SARC028): a multicentre, two‐cohort, single‐arm, open‐label, phase 2 trial. Lancet Oncol. 2017;18:1493‐1501. 28988646 10.1016/S1470-2045(17)30624-1 PMC7939029

[29] Monga V , Skubitz KM , Maliske S , et al. A retrospective analysis of the efficacy of immunotherapy in metastatic soft‐tissue sarcomas. Cancers (Basel). 2020;12:1873. 32664595 10.3390/cancers12071873 PMC7408640

[30] Groisberg R , Hong DS , Behrang A , et al. Characteristics and outcomes of patients with advanced sarcoma enrolled in early phase immunotherapy trials. J Immunother Cancer. 2017;5:100. 29254498 10.1186/s40425-017-0301-y PMC5735899

[31] Adra N , Einhorn LH , Althouse SK , et al. Phase II trial of pembrolizumab in patients with platinum refractory germ‐cell tumors: a Hoosier Cancer Research Network Study GU14‐206. Ann Oncol. 2018;29:209‐214. 29045540 10.1093/annonc/mdx680

[32] Tsimberidou A‐M , Vo HH , Subbiah V , et al. Pembrolizumab in patients with advanced metastatic germ cell tumors. Oncologist. 2021;26:558‐e1098. 33491277 10.1002/onco.13682 PMC8265349

[33] Chovanec M , Mardiak J , Mego M . Immune mechanisms and possible immune therapy in testicular germ cell tumours. Andrology. 2019;7:479‐486. 31169364 10.1111/andr.12656

[34] Necchi A , Bratslavsky G , Corona RJ , et al. Genomic characterization of testicular germ cell tumors relapsing after chemotherapy. Eur Urol Focus. 2020;6:122‐130. 30025711 10.1016/j.euf.2018.07.013

[35] Cierna Z , Mego M , Miskovska V , et al. Prognostic value of programmed‐death‐1 receptor (PD‐1) and its ligand 1 (PD‐L1) in testicular germ cell tumors. Ann Oncol. 2016;27:300‐305. 26598537 10.1093/annonc/mdv574 PMC4751222

[36] Davis KL , Fox E , Merchant MS , et al. Nivolumab in children and young adults with relapsed or refractory solid tumours or lymphoma (ADVL1412): a multicentre, open‐label, single‐arm, phase 1‐2 trial. Lancet Oncol. 2020;21:541‐550. 32192573 10.1016/S1470-2045(20)30023-1 PMC7255545

[37] Geoerger B , Zwaan CM , Marshall LV , et al. Atezolizumab for children and young adults with previously treated solid tumours, non‐Hodgkin lymphoma, and Hodgkin lymphoma (iMATRIX): a multicentre phase 1–2 study. Lancet Oncol. 2020;21:134‐144. 31780255 10.1016/S1470-2045(19)30693-X

[38] Davis KL , Fox E , Isikwei E , et al. A phase I/II trial of nivolumab plus ipilimumab in children and young adults with relapsed/refractory solid tumors: a children's oncology group study ADVL1412. Clin Cancer Res. 2022;28:5088‐5097. 36190525 10.1158/1078-0432.CCR-22-2164 PMC10597535

[39] Scheinberg T , Lomax A , Tattersall M , et al. PD‐1 blockade using pembrolizumab in adolescent and young adult patients with advanced bone and soft tissue sarcoma. Cancer Rep (Hoboken). 2021;4:e1327. 33314769 10.1002/cnr2.1327 PMC8451371

[40] Anderson P , Ghisoli M , Crompton BD , et al. Pilot study of recurrent Ewing's sarcoma management with vigil/temozolomide/irinotecan and assessment of circulating tumor (ct) DNA. Clin Cancer Res. 2023;29:1689‐1697. 36780200 10.1158/1078-0432.CCR-22-2292 PMC10150239

[41] Losa F , Fernández I , Etxaniz O , et al. SEOM‐GECOD clinical guideline for unknown primary cancer (2021). Clin Transl Oncol. 2022;24:681‐692. 35320504 10.1007/s12094-022-02806-x PMC8986666

[42] Pavlidis N , Rassy E , Smith‐Gagen J . Cancer of unknown primary: incidence rates, risk factors and survival among adolescents and young adults. Int J Cancer. 2020;146:1490‐1498. 31144291 10.1002/ijc.32482

[43] Zarkavelis G , Mauri D , Pentheroudakis G . How I treat cancers of unknown primary. ESMO Open. 2019;4:4. 10.1136/esmoopen-2019-000502 PMC6555599 31231571

[44] Raghav KP , Stephen B , Karp DD , et al. Efficacy of pembrolizumab in patients with advanced cancer of unknown primary (CUP): a phase 2 non‐randomized clinical trial. J Immunother Cancer. 2022;10:e004822. 35618285 10.1136/jitc-2022-004822 PMC9125753

[45] Falcone R , Lombardi P , Filetti M , et al. Molecular profile and matched targeted therapy for advanced breast cancer patients. Curr Oncol. 2023;30:2501‐2509. 36826152 10.3390/curroncol30020191 PMC9954949

[46] Fountzilas E , Tsimberidou AM , Vo HH , Kurzrock R . Clinical trial design in the era of precision medicine. Genome Med. 2022;14:101. 36045401 10.1186/s13073-022-01102-1 PMC9428375

[47] Flaherty KT , Gray RJ , Chen AP , et al. Molecular landscape and actionable alterations in a genomically guided cancer clinical trial: National Cancer Institute molecular analysis for therapy choice (NCI‐MATCH). J Clin Oncol. 2020;38:3883‐3894. 33048619 10.1200/JCO.19.03010 PMC7676882

[48] Trédan O , Wang Q , Pissaloux D , et al. Molecular screening program to select molecular‐based recommended therapies for metastatic cancer patients: analysis from the ProfiLER trial. Ann Oncol. 2019;30:757‐765. 30865223 10.1093/annonc/mdz080

[49] Al‐Toubah T , Halfdanarson T , Gile J , Morse B , Sommerer K , Strosberg J . Efficacy of ipilimumab and nivolumab in patients with high‐grade neuroendocrine neoplasms. ESMO Open. 2022;7:100364. 34973511 10.1016/j.esmoop.2021.100364 PMC8728436

