# Supplementary material for: The efficacy of immunotherapy and chemoimmunotherapy in patients with advanced rare tumors: A Turkish oncology group (TOG) study
Source: Cancer Med. 2023 Dec 22;13(1):e6869. doi: 10.1002/cam4.6869 (PMC10809296; doi:10.1002/cam4.6869)
Supplement: Supplementary file 1 — Table S1. [file CAM4-13-e6869-s001.docx]

**Supplementary Table.** The response and disease control rates in individual bone and soft tissue sarcoma subtypes.

|  | | **ORR** | | **DCR** | |
| --- | --- | --- | --- | --- | --- |
|  |  | **Absent** | **Present** | **Absent** | **Present** |
|  |  | **n (%)** | **n (%)** | **n (%)** | **n (%)** |
| **Soft Tissue Sarcoma** | Angiosarcoma | 0 | 1 | 0 | 1 |
|  | ASPS | 1 | 0 | 1 | 0 |
|  | Dendritic Cell Sarcoma | 0 | 1 | 0 | 1 |
|  | Kaposi Sarcoma | 0 | 1 | 0 | 1 |
|  | Leiomyosarcoma | 5 | 1 | 5 | 1 |
|  | PNET | 1 | 0 | 0 | 1 |
|  | Rhabdomyosarcoma | 1 | 0 | 1 | 0 |
|  | Unclassified Sarcoma | 2 | 1 | 1 | 2 |
|  | Uterin Leiomyosarcoma | 1 | 0 | 1 | 0 |
| **Bone Sarcoma** | Chondrosarcoma | 1 | 0 | 1 | 0 |
|  | Chordoma | 1 | 0 | 0 | 1 |
|  | EWS | 4 | 3 | 3 | 4 |
|  | Osteosarcoma | 2 | 0 | 2 | 0 |
